# New species and records of *Pyxine* (Caliciaceae) in China

**DOI:** 10.3897/mycokeys.45.29374

**Published:** 2019-01-29

**Authors:** Mei-Xia Yang, Xin-Yu Wang, Dong Liu, Yan-Yun Zhang, Li-Juan Li, An-Cheng Yin, Christoph Scheidegger, Li-Song Wang

**Affiliations:** 1 Swiss Federal Institute for Forest, Snow and Landscape Research (WSL), Zürcherstrasse 111, CH-8903 Birmensdorf, Zurich, Switzerland Kunming Institute of Botany, Chinese Academy of Sciences Kunming China; 2 University of Bern, Hochschulstrasse 6, 3012 Bern, Switzerland Swiss Federal Institute for Forest, Snow and Landscape Research Zurich Switzerland; 3 Key Laboratory for Plant Diversity and Biogeography of East Asia, Kunming Institute of Botany, Chinese Academy of Sciences, Heilongtan, Kunming, Yunnan 650201, China University of Bern Bern Switzerland; 4 Korean Lichen Research Institute (KoLRI), Sunchon National University, 255 Jungang-Ro, Suncheon, Korea Sunchon National University Suncheon Korea, South

**Keywords:** China, lichenised fungi, new species, phylogeny

## Abstract

In this study, the diversity of *Pyxine* Fr. in China was assessed based on morphological and chemical traits and molecular data are inferred from ITS and mtSSU sequences. Nineteen species were recognised, including three that are new to science (i.e. *P.flavicans* M. X. Yang & Li S. Wang, *P.hengduanensis* M. X. Yang & Li S. Wang and *P.yunnanensis* M. X. Yang & Li S. Wang) and three records new to China were found (i.e. *P.cognata* Stirt., *P.himalayensis* Awas. and *P.minuta* Vain.). *Pyxineyunnanensis* is diagnosed by the small size of the apothecia, a white medulla of the stipe and the presence of lichexanthone. *Pyxineflavicans* is characterised by broad lobes, a pale yellow medulla of the stipe and the presence of atranorin. *Pyxinehengduanensis* can be distinguished by its pale yellow medulla, marginal labriform soralia and the absence of atranorin. Detailed descriptions of each new species are presented, along with a key to the known species of *Pyxine* in China.

## Introduction

The lichen genus *Pyxine* was first established by Fries (1825). Molecular data support the placement of *Pyxine* in a clade of taxa that were previously placed in Physciaceae and the circumscription of the family has thus changed to Caliciacese (Wedin and Grube 2002; Crespo et al. 2004; Gaya et al. 2012; Prieto and Wedin 2017). *Pyxine* is characterised by an adnate foliose thallus, an internal stipe colour of apothecia, dark brown hypothecium and generally two-celled brown ascospores (Awasthi 1982; Elix 2009; Kalb 1987; Kalb 2004). The genus *Pyxine* consists of approximately 70 species. Most species are pantropical to subtropical and a few species extend into temperate or oceanic regions (Elix 2009; Mongkolsuk et al. 2012; Kalb 1987; Moberg 1983; Wei and Hur 2007).

Regional studies on this genus have been carried out in Australia (Elix 2009), Brazil (Aptroot et al. 2014), India (Awasthi 1982; Nayaka et al. 2013), Thailand (Mongkolsuk et al. 2012) and North and Central America (Imshaug 1957; Jungbluth and Marcelli 2011). Prior to this study, 13 species have been reported in China, including *Pyxineberteriana*, *P.cocoes*, *P.consocians*, *P.copelandii*, *P.coralligera*, *P.endochrysina*, *P.limbulata*, *P.meissnerina*, *P.microspora*, *P.petricola*, *P.philippina*, *P.sorediata* and *P.subcinerea* (Hu and Chen 2003; Obermayer and Kalb 2010; Wei 1991).

Although many studies have been conducted, few molecular phylogenetic analyses have been completed (Gaya et al. 2012; Schmull et al. 2011; Prieto and Wedin 2017). In this study, morphological, chemical and molecular phylogenetic analyses were combined in order to re-evaluate the species composition and phylogenetic relationship of this genus in China. In our study, 31 sequences were newly generated from freshly collected specimens.

## Methods

### Morphological and chemical analyses

The specimens examined in this study were collected from the Hengduan Mountains region, Taiwan, Zhejiang, Hainan et al. from 1941 to 2016 and deposited in KUN-L (325 specimens) and in the Institute of Microbiology (HMAS-L, 5). Morphological characteristics were studied using a dissecting microscope (Nikon SMZ745T) and a light microscope (Nikon Eclipse Ci-S; Nikon Instruments, Tokyo Japan). Sections were made with a razor blade under a dissecting microscope and anatomical characteristics were examined and measured using a micrometer under light microscopy. Ten measurements each of the thallus, apothecia and ascospore dimensions were taken from a single apothecium per specimen and the ranges of these measurements, from smallest to largest, are presented in this study. The lichen secondary metabolites were analysed using spot reactions and thin-layer chromatography in a solvent C system, according to Orange et al. (2001).

### DNA extraction and sequencing

Total genomic DNA was extracted from dried herbarium specimens using AxyPrep Multisource Genomic DNA Miniprep Kit 50-prep (Qiagen) according to the manufacturer’s instructions. ITS (nrDNA ITS1-5.8S-ITS2) and mtSSU (mitochondrial small subunit rDNA) were amplified by polymerase chain reactions (PCR) using the primer pairs ITS1F (Gardes and Bruns 1993), ITS4 (White et al. 1990) and mtSSU1/mtSSU2R (Zoller et al. 1999).

Amplifications were performed in a 25 μl volume comprising 12.5 μl of 2× MasterMix (TapDNA Polymerase, 0.1 units/μl; technologies Co. Ltd), 1.0 μl of each primer, 8.5 μl ddH_2_O and 2 μl DNA. Conditions for the PCR were: initial denaturation at 94 °C for 4 min, 34 cycles at 94 °C for 1 min, 54 °C for 1 min and 72 °C for 1.5 min, with a final extension at 72 °C for 10 min. PCR products were sequenced in an ABI3730X using amplification primers manufactured by Tsingke (Kunming, China).

ITS and mtSSU sequences were assembled with Seqman 7.0 (DNAStar) and manually edited using Mega6. DNA sequences were aligned with MAFFT version 7 with default parameters (Katoh et al. 2005) via the online resource (http://mafft.cbrc.jp/alignment/server/index.html).

### Phylogenetic analyses

Maximum likelihood (ML) and Bayesian inference (BI) were conducted based on the two gene fragments combining ITS and mtSSU. The best-fitting substitution model was determined using MrModeltest 2.3 (Nylander 2005) and PAUP*4b10 (Swofford 2003), where the AIC values were calculated using JModelTest 3.7 (Posada 2008). ML analyses were performed using RAxML7.0.4 (Stamatakis 2006) with default settings (GTR) and support values were inferred from the 70% majority-rule tree based on 1000 non-parametric bootstrap pseudo-replicates. The Bayesian analyses were performed using MrBayes v3.1.2 (Huelsenbeck and Ronquist 2001) with 2,000,000 generations and four incrementally heated chains. MCMC (Markov Chain Monte Carlo) analysis started from a random tree that was sampled every 1000^th^ generation, with the first 10% of trees discarded as burn-in. A majority-rule consensus tree was constructed from the remaining trees to estimate posterior probability (PP), with values greater than or equal to 0.95 considered indicative of strong support. Tracer v1.6 (Rambaut and Drummond 2003) was used to ensure that stationarity was achieved by checking whether the log-likelihood values of sample points reached a stable equilibrium. Phylogenetic trees were visualised using the programme FigTree 1.4.0 (Rambaut 2012). *Physciadubia* and *Dirinariaapplanata* were selected as outgroups.

## Results

Nineteen species were recognised, including three that are new to science (i.e. *Pyxineflavicans* M. X. Yang & Li S. Wang, *P.hengduanensis* M. X. Yang & Li S. Wang and *P.yunnanensis* M. X. Yang & Li S. Wang) and three records new to China were found (i.e. *P.cognata* Stirt., *P.himalayensis* Awas. and *P.minuta* Vain.). Of the 39 sequences that were included in the phylogenetic analyses, 31 were newly generated (Table 1). A phylogenetic analysis using ITS and mtSSU sequences revealed 15 species. We were unable to obtain sequences from *P.copelandii*, *P.coralligera*, *P.microspora* and *P.philippina*, but the Chinese specimens agreed morphologically and chemically with the current circumscription of these species (Hu and Chen 2003; Obermayer and Kalb 2010; Wei 1991).

**Table 1.** Specimen information and GenBank accession numbers for taxa used in this study. Newly obtained sequences are shown in bold.

**Taxa**	**Locality**	**Voucher specimens**	**Accession Number**	
			**ITS**	**mtSSU**
*Pyxinesorediata* 1	China: Yunnan	KUN 12-36993	**KY611891**	**KY751398**
*P.sorediata* 2	China: Yunnan	KUN 15-48546	**KY611892**	**KY751399**
*P.sorediata* 3	Sweden	Wetmore 91254 (UPS)	JX000111	–
			–	KX512973
*P.hengduanensis* 1	China: Yunnan	KUN 15-48082	**KY611889**	**KY751396**
*P.hengduanensis* 2	China: Yunnan	KUN14-43258	**KY611890**	**KY751397**
*P.endochrysina* 1	China: Xizang	KUN 14-46462	**KY611887**	**KY751394**
*P.endochrysina* 2	China: Xizang	KUN 14-46439	**KY611888**	**KY751395**
*P.limbulata* 1	China: Taiwan	KUN 15-49117	**KY611885**	**KY751392**
*P.limbulata* 2	China: Taiwan	KUN 15-49153	**KY611886**	**KY751393**
*P.himalayensis* 1	China: Yunnan	KUN 12-36055	**KY611881**	**KY751388**
*P.himalayensis* 2	China: Xizang	KUN 14-46410	**KY611882**	**KY751389**
*P.flavicans* 1	China: Yunnan	KUN 14-43995	**KY611883**	**KY751390**
*P.flavicans* 2	China: Yunnan	KUN 15-48196	**KY611884**	**KY751391**
*P.meissnerina* 1	China: Yunnan	KUN 12-34386	**KY611877**	**KY751384**
*P.meissnerina* 2	China: Yunnan	KUN 12-34377	**KY611878**	**KY751385**
*P.consocians* 1	China: Yunnan	KUN 15-49942	**KY611879**	**KY751386**
*P.consocians* 2	China: Yunnan	KUN 15-47417	**KY611880**	**KY751387**
*P.petricola* 1	China: Yunnan	KUN 13-40715	**KY611875**	**KY751382**
*P.petricola* 2	China: Sichuan	KUN 13-39419	**KY611876**	**KY751383**
*P.cocoes* 1	China: Taiwan	KUN 15-49457	**KY611874**	**KY751381**
*P.minuta* 1	China: Yunnan	KUN 13-40695	**KY611872**	**KY751379**
*P.minuta* 2	China: Yunnan	KUN 13-40630	**KY611873**	**KY751380**
*P.yunnanensis* 1	China: Yunnan	KUN 13-41372	**KY611870**	**KY751377**
*P.yunnanensis* 2	China: Yunnan	KUN 13-40596	**KY611871**	**KY751378**
*P.berteriana* 1	China: Yunnan	KUN 15-47921	**KY611868**	**KY751375**
*P.berteriana* 2	China: Yunnan	KUN 14-43730	**KY611869**	**KY751376**
*P.subcinerea* 1	China: Taiwan	KUN 15-48998	**KY611866**	**KY751373**
*P.subcinerea* 2	China: Taiwan	KUN 15-49012	**KY611867**	**KY751374**
* P. subcinerea *	USA	NC 27708	HQ650705	–
	Spain	MAF9852	–	AY464080
*P.cognata* 1	China: Yunnan	KUN 14-43569	**KY611864**	**KY751371**
*P.cognata* 2	China: Yunnan	KUN 13-40767	**KY611865**	**KY751372**
P.berterianavar.himalaica 1	China: Yunnan	KUN 14-43571	**KY611862**	**KY751369**
P.berterianavar.himalaica 2	China: Yunnan	KUN 13-40706	**KY611863**	**KY751370**
* Dirinaria applanata *	India	–	EU722342	–
	Spain	MAF 9854	–	AY464079
* Physcia dubia *	Finland	T. Ahti 69359	JQ301695	–
			–	JQ301536

**Figure 1.**
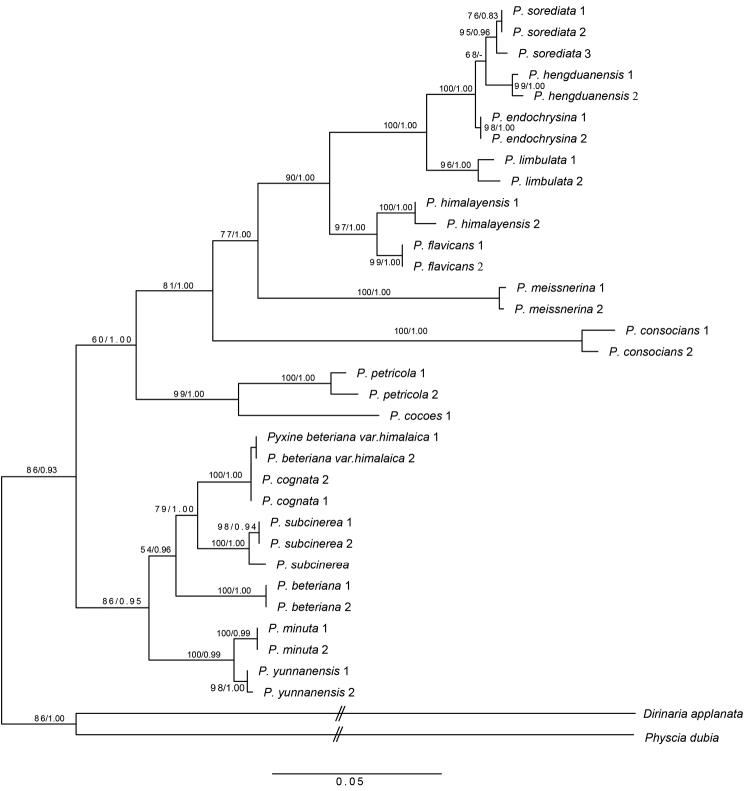
Phylogenetic relationship of *Pyxine* species occurring in China inferred from ITS and mtSSU sequences using maximum likelihood (values refer to ML bootstrap frequencies and Bayesian posterior probabilities).

The ITS and mtSSU datasets were analysed separately and concatenated; both parsimony and Bayesian trees of ITS vs. mtSSU were congruent. A maximum likelihood phylogenetic tree was inferred from the combined dataset of ITS and mtSSU (Fig. 1). The monophyly of each species and the phylogenetic relationships between species were well supported (Fig. 1; MLBS > 90%, PP > 0.95). Specifically, the three new species were all monophyletic with a high support value: *Pyxineyunnanensis* (MLBS = 97%, PP = 1.00), *P.flavicans* (MLBS = 99%, PP = 0.99) and *P.hengduanensis* (MLBS = 98%, PP = 1.00).

Species of *Pyxine* were separated into two main clades, as inferred from the phylogenetic tree with strong support (Fig. 1). The ten species in Clade 1 are all characterised by the presence of soralia or isidia on the thallus, whereas the five species in Clade 2 contain lichexanthone and lack soralia and isidia. The two species *P.petricola* and *P.cocoes* are characterised by the presence of both lichexanthone and soralia.

## Taxonomic treatment

Nineteen *Pyxine* species were confirmed in China, including three species new to science and three species hereby newly reported for the country, based on the following characteristics: presence of isidia and soredia, colour of the medulla, main compounds, reaction of K on the internal stipe of apothecia, nature of the substrate and colour of the thallus.

### New species

#### Pyxine flavicans

Taxon classificationFungiTeloschistalesPhysciaceae

M. X. Yang & Li S. Wang sp. nov.

819956

Figure 2

##### Holotype.

CHINA, YUNNAN PROVINCE, Nujiang Perf., Chide Vil., 1916 m elevation, 27°42'32"N, 98°43'19"E, on *Juglans*, 4 Aug 2015, *L. S. Wang* et al. KUN-L15-48196. GenBank accession No.: ITS = KY611884, mtSSU = KY751391.

##### Description.

Thallus 5–9 cm wide, attached to closely adnate. Lobes radiating, plane to convex, but often slightly concave towards the tips, (0.5) 1–3 (4) mm wide, subround at the apices. Upper surface white-grey to celadon, sparsely pruinose at the lobe tips or epruinose, isidia and soredia absent. Medulla pale yellow above, white below. Lower surface black in the centre, paler towards the margin; rhizines dense, furcate. Apothecia common, (0.5) 0.8–1.5 (2) mm wide, constricted at base, plane to possibly convex; margin black. Hymenium height 80–120 μm; hypothecium light brown to brown, internal stipe K– pale yellow to yellow; spores brown, two-celled, 18–20 × 6–8 μm. Upper cortex K+ yellowish, UV–; medulla K–, C–; containing atranorin, chloroatranorin (minor), zeorin and unknown terpenes.

##### Habitat and distribution.

Growing on bark of *Quercus* and *Picea* spp. and on rocks around 1916–4000 m elevation in semi-arid environments; only known from south-western China.

##### Etymology.

The epithet *flavicans* refers to the yellow medulla and internal stipe of the apothecia.

##### Notes.

*Pyxineflavicans* is characterised by flat corticated yellowish-grey to brownish-grey thalli, a constricted base, a pale yellow medulla and the presence of atranorin.

This species resembles *P.berteriana* in terms of lobe size, saxicolous habitat and internal stipe, but the latter has a yellow to yellowish-orange medulla and produces lichexanthone (Hu and Chen 2003). *Pyxineflavicans* is similar to *P.australiensis* Kalb regarding the absence of soredia and isidia and both species are frequently lignicolous but occasionally grow on rocks. However, *P.flavicans* differs from *P.australiensis* in having marginal and laminal pseudocyphellae, lichexanthone and a white medulla in the stipe (Elix 2009). *Pyxineflavicans* is similar to *P.himalayensis* in terms of the type of apothecia and lack of lichexanthone. However, *P.himalayensis* has a colourless internal stipe.

##### Selected specimens examined (KUN).

CHINA: SICHUAN PROVINCE: Muli Co., 2850 m elev., on *Pinusyunnanensis*, 23 Aug 1983, *L. S. Wang* 83-1869(A); XIZANG PROVINCE: Chayu Co., along the road from Muruo Vil. to Bingzhongluo, 3833 m elev., 28°35.781'N, 98°06.404'E, on *Pinusarmandii*, 26 Sep 2014, *L. S. Wang* et al. 14-46763; YUNNAN PROVINCE: Jianchuan Co., Shibao Mt., 2620 m elev., 26°22.920'N, 99°49.811'E, on bark, 24 Jun 2014, *L. S. Wang* et al. 14-43995; Nujiang Co., Chide Vil., 1916 m elev., 27°42'32.40"N, 98°43'18.59"E, on *Juglans*, 4 Aug 2015, *L. S. Wang* et al. 15-48196.

**Figure 2.**
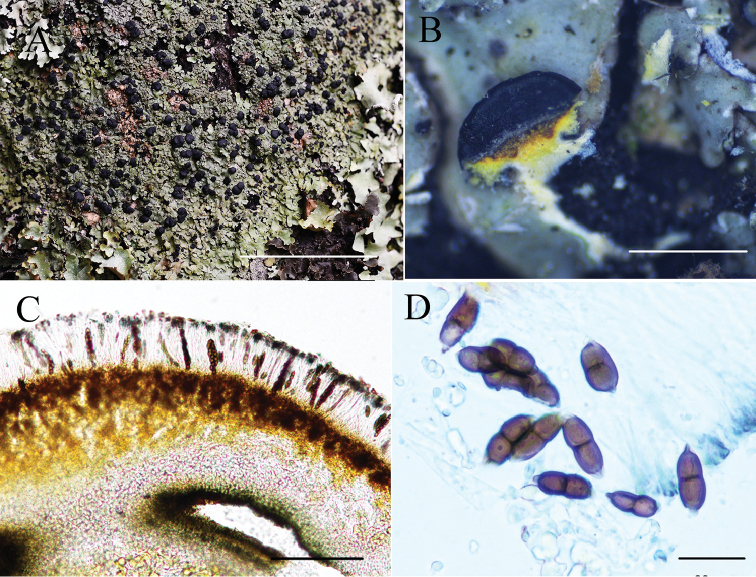
*Pyxineflavicans* (KUN-L 15-48196) photographed by Li-Song Wang and Meixia Yang. **A** habit **B** yellow internal stipe of apothecia **C** hymenium **D** ascospores from GAW (glycerine:alcohol:water=1:1:1). Scale bars: 5 cm (**A**); 0.5 cm (**B**); 100 μm (**C**); 20 μm (**D**).

#### Pyxine hengduanensis

Taxon classificationFungiTeloschistalesPhysciaceae

M. X. Yang & Li S. Wang sp. nov.

819957

Figure 3

##### Holotype.

CHINA, YUNNAN PROVINCE, Nujiang Pref., Dizhengdang Vil., 1858 m elevation, 28°05'00.86"N, 98°19'39.97"E, on bark, 2 Aug 2015, *L. S. Wang* et al. KUN-L 15-48082. GenBank accession No.: ITS = KY611889, mtSSU = KY751396.

##### Description.

Thallus corticolous, 4–9 cm wide, firmly to loosely adnate to substrate. Lobes linear, compact, imbricate to discrete, (0.5) 1–2.5 mm wide, upper cortex plane but often slightly concave towards the tips; pseudocyphellae linear, marginal; upper surface grey to greyish-green, lower-side black; rhizines dense, squarrosely branched. Soralia marginal, labriform; soredia grey to bluish-grey, powdery to granular. Medulla pale yellow. Dactyls and isidia absent. Apothecia absent. Upper cortex K+ yellowish, UV–; medulla K–, C–; containing chloroatranorin (minor) and unknown terpenes.

##### Habitat and distribution.

Growing on bark of *Quercus* and *Alnus* spp. Range 1700 –3060 m elevation in semi-arid environments; known only from Yunnan, Sichuan and Xizang in China.

##### Etymology.

The epithet *hengduanensis* refers to the type locality of the species, the Hengduan Mountains region.

##### Notes.

*Pyxinehengduanensis* is characterised by a corticolous habit, yellowish-grey to greyish-green thallus, marginal labriform soralia, pale yellow medulla and the absence of atranorin. *Pyxinehengduanensis* is most closely related to *P.sorediata*, as inferred from the phylogenetic tree (Fig. 1); *P.sorediata* is also corticolous but has a yellow or yellow-orange medulla and soralia that develop marginally from fissures and then become laminal and orbicular (Elix 2009), while *P.hengduanensis* has marginal labriform soralia developing from the centre of the pseudocyphellae, grey to bluish-grey soredia and a pale yellow medulla. *Pyxinehengduanensis* also resembles *P.retirugella* Nyl. (Elix 2009) in the marginal and laminal pseudocyphellae, but it differs in having white or creamy and K+ yellow turning red medulla and norstictic acid as the main compound (Mongkolsuk et al. 2012).

##### Selected specimens examined (KUN).

CHINA: SICHUAN PROVINCE: Dukou Co., Yanbian Vil., Shibao Mt., 2900 m elev., 29 Jun 1983, *L. S. Wang* 83-635; Nanping Co., Jiuzhaigou, 2000 m elev., on *Pinus*, 23 Sep 1986, *L. S. Wang* 86-2591. XIZANG PROVINCE: Linzhi Co., 3060 m elev., 29°50'249"N, 94°44'728"E, on *Quercus* spp. 20 Aug 2007, *L. S. Wang* et al. 07-28389; YUNNAN PROVINCE: Luquan Co., 30 km from Sapanying Co. to Zehei Co., 2540 m elev., 26°04'24.53"N, 102°36'19.15"E, on moss, 19 Apr 2014, *L. S. Wang* et al. 14-43258; Lufeng Co., Heijin Vil., 1800 m elev., 25°20'146"N, 102°05'835"E, on bark, 1 May 2009, *L. S. Wang* 09-30247.

**Figure 3.**
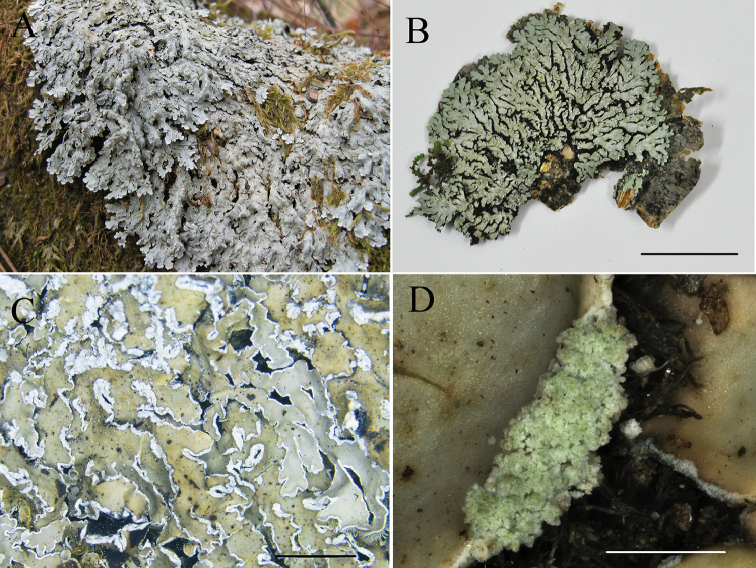
*Pyxinehengduanensis*: **A** (KUN-L 09-30247) photographed by Li-Song Wang, *in situ* at the type locality **B–D** (KUN-L 15-48082), photographed by Mei-xia Yang **B** Thallus **C** upper surface of thallus **D** marginal labriform soralia. Scale bars: 2 cm (**B**); 5 mm (**C**); 0.5 mm (**D**).

#### Pyxine yunnanensis

Taxon classificationFungiTeloschistalesPhysciaceae

M. X. Yang & Li S. Wang sp. nov.

819958

Figure 4

##### Holotype.

CHINA, YUNNAN PROVINCE, Yongren Co., Lagu Vil., 1050 m elevation, 26°23.239'N, 101°25.120'E, on rock, 4 Dec 2013, *L. S. Wang* et al. KUN-L 13-41372. GenBank accession No.: ITS = KY611870, mtSSU = KY751377.

##### Description.

Thallus saxicolous, up to 7 cm in diam., closely appressed to the substrate. Lobes radiating, irregularly branched, plane to slightly concave, 0.2–1.0 mm wide, subround to truncate at the apices. Upper surface pale grey to yellowish-grey, sparsely pruinose at the lobe tips or epruinose. Lower surface brownish-black, rarely pale brown, rhizines indistinct, sparse to moderately abundant, brownish-black to black. Isidia and soredia absent. Medulla pale yellow in the upper part, white in the lower part. Apothecia abundant, 0.2–0.8 mm wide, constricted at base, plane to possibly convex; margin black. Hymenium height 80–120 μm; hypothecium light brown to brown, internal stipe white; spore brown with two cells, 10–15 × 4–7 μm. Upper cortex K+ yellowish, UV+ yellow; medulla K–, C–; containing lichexanthone, chloroatranorin (minor), zeorin and unknown terpenes (detected by TLC).

##### Habitat and distribution.

Growing on rocks around 1050–1650 m elevation in secondary forests in a dry to semi-arid environment; known only from Yunnan.

##### Etymology.

The epithet *yunnanensis* refers to the province of the type locality of the species.

##### Notes.

*Pyxineyunnanensis* is characterised by small and saxicolous thalli, rather small narrow apothecia (up to 0.8 mm in diam.), a white internal stipe and the presence of lichexanthone. *Pyxineminuta* Vain. (up to 3 cm in diam.) resembles *P.yunnanensis* (up to 7 cm in diam.) in its small thalli and the presence of lichexanthone, but differs in that its internal stipe is absent or indistinct and it has a white medulla (Awasthi 1982). *Pyxinepyxinoides* (Müll. Arg.) Kalb and *P.elixii* Kalb also grow on rocks, but *P.pyxinoides* differs from *P.yunnanensis* in that it has a white medulla, an indistinct internal stipe of the apothecia and smaller ascospores (10–15 × 4–7 μm) than those of *P.pyxinoides* (10–16 × 4.5–8.0 μm). *Pyxineelixii* can be distinguished by its orange medulla and lack of lichexanthone (Elix 2009).

*Pyxineyunnanensis* is closely related to *P.berteriana* in that they have a similar type and size of apothecia and lichexanthone is present, but *Pyxineberteriana* differs in that it occurs in incorticolous habitat and has a yellow medulla and a yellow medulla of the stipe.

##### Selected specimens examined (KUN).

CHINA: YUNNAN PROVNCE: Yongsheng Co., Dongjiang of Renhe Town, 1130 m elev., 26°20.448'N, 101°06.908'E, on rock, 7 Dec 2013, *L. S. Wang* et al. 13-41413; Shawan Village of Renhe Town, 1160 m elev., 26°19.449'N, 101°05.200'E, 7 Dec 2013, *L. S. Wang* et al. 13-40643, 13-40694, 13-40686, 13-40641, 13-40684; Lijiang City, east of Jinan Bridge, 1310 m elev., 26°47.725'N, 100°25.640'E, on rock, 8 Dec 2013, *L. S. Wang* et al. 13-40596.

**Figure 4.**
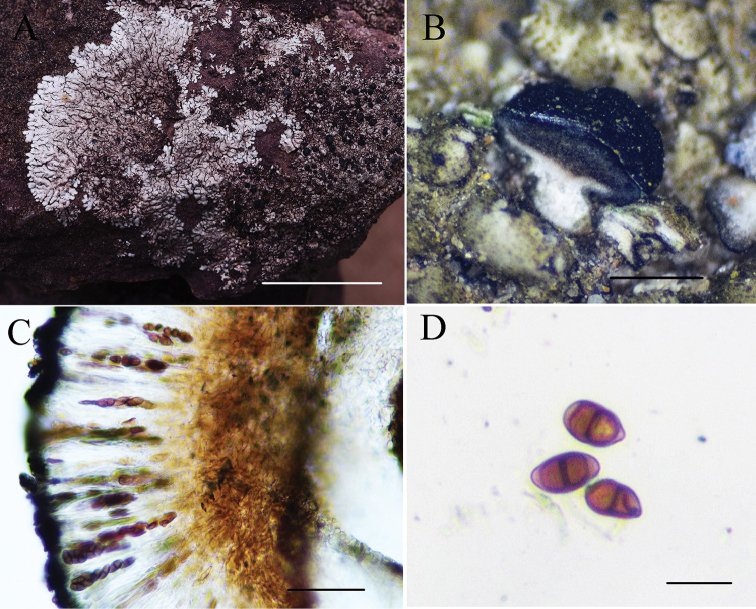
*Pyxineyunnanensis*: **A** (KUN-L 09-30247) photographed by Li-Song Wang, in situ at the type locality **B–D** (KUN-L 13-41372) photographed by Mei-xia Yang **B** white internal stipe of apothecia **C** hymenium **D** ascospores from GAW (glycerine:alcohol:water=1:1:1). Scale bars: 1 cm (**A**); 1 mm (**B**); 50 μm (**C**); 10 μm (**D**).

### New records

#### Pyxine cognata

Taxon classificationFungiTeloschistalesPhysciaceae

Stirt

= Pyxineberterianavar.himalaica D.D. Awasthi

##### Description.

Upper surface white to whitish-grey or grey-brown; isidia and soredia absent; medulla orange-yellow to orange; lower surface black in the centre, paler towards the margin; apothecia common, (0.3) 0.5–1.0 (1.5) mm wide; internal stipe upper part orange, K+ purple, P–; lower part yellow or much paler than upper part, K–, P–. Upper cortex K–, UV+ yellow, medulla K– or K+ pale red, C–, P– or P+ orange; containing lichexanthone (major), triterpenes, unknown pigment (minor) (detected by TLC).

##### Habitat and distribution.

Growing on bark of *Quercus* and *Juglans* spp. Range 1090–2230 m elevation in semi-arid environments. Worldwide distribution: India (Awasthi 1982), Brazil (Aptroot 2014), Thailand (Mongkolsuk et al. 2012) and Australia (Elix 2009); newly recorded in China.

##### Notes.

Pyxineberterianavar.himalaica was described by Awasthi (1982) as a variety based on the pale yellow to yellow medulla and a narrow distribution from the Himalayan region and central India. *Pyxinecognata* is very similar to P.berterianavar.himalaica in the presence of lichexanthone, the pigmented medulla and the lack of isidia and soredia. However, *Pyxinecognata* is distinguished by a faint pruina on the lobe tips, deep yellow to rust coloured medulla and slightly larger spores, as well as for being widely distributed in tropical regions. Therefore, the morphological and ecological differences between these two species are minor. In this study, we collected specimens of both species and found that they have a similar ecology and distribution pattern. Phylogenetic analysis inferred that Pyxineberterianavar.himalaica is clustered with *P.cognata* with a high support value (MLBS = 100%, PP = 1.00). Based on the combination of molecular, morphological and ecological information, we propose P.berterianavar.himalaica as a synonym for *P.cognata*.

*Pyxinecognata* is most similar to *P.berteriana* in that it contains lichexanthone, lacks isidia and soredia and has a pigmented medulla; however, *P.cognata* can be distinguished by the presence of lichexanthone in the cortex, an orange medulla and an orange-yellow internal stipe of apothecia with K+ purple. In comparison, *P.berteriana* has a pale yellow to yellow medulla and the internal stipe is pale yellow to yellow. (Kalb 1987). Despite the broad similarities, these species are not closely related; *P.cognata* seems to share a unique ancestor with *P.subcinerea*. *Pyxinesubcinerea* differs in that it has marginal soralia and obscurascens-type apothecia (Elix 2009). (Fig. 1).

##### Selected specimens examined (KUN).

CHINA: SICHUAN PROVINCE: Miyi Co., Malong north slope, 2100 m elev., on *Carya* spp., 5 Jul 1983, *L. S. Wang* 83-698; Dukou Co., Dabaoding, 1900 m elev., 21 Jun 1983, *L. S. Wang* 83-212. YUNNAN PROVINCE: Yuanmou Co. Langbapu Forest Soil, 1612 m elev., 25°41'01.76"N, 101°41'25.78"E, on branch, 21 Apr 2014, *c*14-43569, 14-43539; Yongren Co., from Menghu to Wanma, 1543 m elev., 26°13'45.15"N, 101°25'56.86"E, 3 Dec 2013, *L. S. Wang* et al. 13-40767.

#### Pyxine himalayensis

Taxon classificationFungiTeloschistalesPhysciaceae

Awas

##### Description.

Thallus grey-white, soredia and isidia absent; medulla yellow to orange-yellow; apothecia common, laminal, constricted at base, up to 2 mm in diam.; internal stipe colourless, K–, hypothecium 50–80 μm thick, spores brown, 15–25 × 6–9 μm. Upper cortex K+ yellow, UV–, medulla K–, C–, P–; containing atranorin (major), +/– zeroin, triterpense.

##### Habitat and distribution.

Growing on bark of *Rhododendron*, *Quercus*, *Alnus*, *Juglans*, *Sophora*, *Lonicera* and *Lyonia* spp. and rarely on rocks, at elevations of 1330–3600 m in semi-arid environments. Worldwide distribution: India (Awasthi 1982) and added here to the flora of China.

##### Notes.

*Pyxinehimalayensis* is distinctive for having lobes 1.5–3.0 mm wide, an orange medulla and a lack of isidia and soredia, lichexanthone and norstictic acid. *Pyxinehimalayensis* was first described by Awasthi (1982) and it is characterised by an orange medulla and colourless internal stipe of apothecia. The closely related *Pyxinelimbulata* is described as having a yellow medulla and a brown internal stipe (Hu and Chen 2003). There are 24 specimens of this species in the KUN-L. The phylogenetic analysis of ITS and mtSSU sequences confirm that these are independent species.

##### Selected specimens examined (KUN).

CHINA: SICHUAN PROVINCE: Dukou City, Shibao Mt., 2800 m elev., 29 Jun 1983, *L. S. Wang* 83-628; Yuanyang Co., Bailing commune, 3100 m elev., on *Quercus*, 11 Aug 1983, *L. S. Wang* 83-1508; 3250 m elev., on stone, 10 Aug 1983, *L. S. Wang* 83-1483; Muli Co., Yazui forest farm, on *Quercus*, 3000 m elev., 20 Aug 1983, *L. S. Wang* 83-1589, 83-1596; Donglang, 3000 m elev., on bark, 10 Sep 1983, *L. S. Wang* 83-2220. XIZANG PROVINCE: Bomi Co., Gang vil., 2688 m elev., 29°52.983'N, 095°33.593'E, on branch of *Populusyunnanensis*, 20 Sep 2014, *L. S. Wang* et al. 14-46203, 14-46162. YUNNAN PROVINCE: Luquan Co., 30 km from Sapanying Co. to Zehei Co., 2540 m elev., 26°04'24.53"N, 102°36'19.15"E, on *Quercus*, 19 Apr 2014, *L. S. Wang* et al. 14-43218, 14-43204; Luquan Co., Zhongcun Vil., 2350 m elev., 25°20'146"N, 102°05'835"E, on bark of *Quercus*, 1 May 2009, *L. S. Wang* 09-30279.

#### Pyxine minuta

Taxon classificationFungiTeloschistalesPhysciaceae

Vain

##### Description.

*Pyxineminuta* is characterised by narrow lobes, centrally subcrustaceous, saxicolous thalli, a whitish-grey or grey-brown upper surface; brownish-black lower surface with black and simple rhizines, a lack of isidia and soredia and a white or whitish stramineous medulla. Apothecia common, 0.5–1.5 mm wide; internal stipe absent or not distinct. Upper cortex K+ yellowish, UV+ yellow, medulla K–, C–; containing lichexanthone (major) and terpenoids (detected by TLC).

##### Habitat and distribution.

Growing on bark of *Quercus* spp. or rock around 1090–2230 m elevation in semi-arid environments. Worldwide distribution: India (Awasthi 1982), Australia (Rogers 1986) and newly recorded in China.

##### Notes.

There is some confusion in the classification of *Pyxineminuta* and *P.pyxinoides*. *Pyxineminuta* is characterised by narrow lobes, an absent or indistinct internal stipe, small spores (11–16 (18) × 5–7 μm) and a white medulla. Based on the world key to *Pyxine* species with lichexanthone (Aptroot et al. 2014; Kalb 1987; Huneck et al. 1987), the characteristics of *Pyxinepyxinoides* are: Thallus without isidia, pustules or soredia, usually with apothecia; Medulla yellow, ochraceous or salmon; apothecium margin black, not thalline; apothecium without a clear stipe; one TLC run of a portion of the thallus without apothecia showed traces of a substance running like norstictic acid (Obermayer and Kalb 2010); neotropical. We did not find any specimens of *P.pyxinoides* in our collections.

##### Selected specimens examined (KUN).

CHINA: SICHUAN PROVINCE: Dukou Co., Dabaoding, 1950 m elev., 21 Jun 1983, *L. S. Wang* 83-206. YUNNAN PROVINCE: Yongsheng Co., Shawan village of Renhe town, 1160 m elev., 26°19.449'N, 101°05.200'E, on rock, 7 Dec 2013, *L. S. Wang* et al. 13-40630, 13-40695; Yongren Co., Lagu village, 1050 m elev., 26°23.239'N, 101°25.120'E, on rock, 4 Dec 2013, *L. S. Wang* et al. 13-41380; Jinggu Co., on the way to Zhenyuan, 1800 m elev., 21 Aug 1994, *L. S. Wang* et al. 94-14247.

### Key to the species of the genus *Pyxine* in China

**Table:** 

1	Thallus UV+, lichexanthone present	**2**
–	Thallus UV–, lichexanthone absent	**12**
2	Thallus with vegetative propagules	**3**
–	Thallus lacking vegetative propagules	**7**
3	Thallus with soredia	**4**
–	Thallus with isidia	**5**
4	Medulla yellow	*** P. subcinerea ***
–	Medulla white	*** P. cocoes ***
5	Medulla yellow; isidia dactyliform	*** P. endochrysina ***
–	Medulla white; isidia cylindrical	**6**
6	Norstictic acid present	*** P. consocians ***
–	Norstictic acid absent	*** P. coralligera ***
7	Atranorin present	*** P. cognata ***
–	Atranorin absent	**8**
8	Medulla pale yellow to yellow	*** P. berteriana ***
–	Medulla white	**9**
9	Internal stipe of apothecia absent or indistinct	**10**
–	Internal stipe of apothecia well developed	**11**
10	Norstictic acid present, as well as other triterpenes	*** P. microspora ***
–	Norstictic acid absent	*** P. minuta ***
11	Internal stipe of apothecia brown, K+ red violet	*** P. petricola ***
–	Internal stipe of apothecia white, K–	*** P. yunnanensis ***
12	Thallus with soralia	**13**
–	Thallus lacking vegetative propagules	**16**
13	Medulla white, soralia laminal; norstictic acid present	*** P. copelandii ***
	Medulla yellow; soralia marginal; norstictic acid absent	**14**
14	Atranorin absent; soralia labriform	*** P. hengduanensis ***
–	Atranorin present; soralia granular, laminal or orbicular	**15**
15	Lobe margin without pseudocyphellae; soredia yellow	*** P. meissnerina ***
–	Lobe margin with intermittent pseudocyphellae; soredia grey to bluish-grey	*** P. sorediata ***
16	Medulla yellow	**17**
–	Medulla white	*** P. philippina ***
17	Internal stipe of apothecia colourless	*** P. himalayensis ***
–	Internal stipe of apothecia brown or yellow	**18**
18	Internal stipe brown, K+ red violet	*** P. limbulata ***
–	Internal stipe of apothecia pale yellow; upper medulla yellow, lower medulla white	*** P. flavicans ***

## Supplementary Material

XML Treatment for Pyxine flavicans

XML Treatment for Pyxine hengduanensis

XML Treatment for Pyxine yunnanensis

XML Treatment for Pyxine cognata

XML Treatment for Pyxine himalayensis

XML Treatment for Pyxine minuta
